# Microbiological Analysis of Primary Molars Restored with Stainless Steel Crowns Compared to Healthy Molars

**DOI:** 10.3390/microorganisms13061294

**Published:** 2025-05-31

**Authors:** Andrea Rubio, Tanya Pereira, Juan Ramón Boj, Teresa Vinuesa

**Affiliations:** 1 Department of Dentistry, Faculty of Medicine and Health Sciences, University of Barcelona, 08907 L’ Hospitalet de Llobregat, Spain; 2 Department of Pathology and Experimental Therapeutics, Faculty of Medicine and Health Sciences, IDIBELL Institute, University of Barcelona, 08907 L’ Hospitalet de Llobregat, Spain

**Keywords:** stainless-steel crowns, primary molars, gingival health, oral microbiota, microbial adhesion

## Abstract

One of the best restorative treatment options for large carious lesions is the placement of stainless-steel crowns (SSC), but there are few studies evaluating if there is any change in the microbiota in teeth restored with SSCs. In order to asses if any difference exists, 33 children between 4 and 10 years were studied. One primary molar restored with an SSC as well as one healthy primary molar were selected from each child. Subgingival plaque was collected with a curette and cultured on horse blood agar (Columbia) and selective and nonselective media. A quantitative analysis was performed by means of the counting of the colony-forming units per milliliter (cfu/mL) grown in the nonselective media and compared with the bacterial load measured by means of a 16S qPCR with bacterial universal primers. A descriptive statistical analysis was performed to evaluate the results. No significant differences were observed in the total 16S qPCR according to sample type. Streptococci were observed in all the studied children. *Porphyromonas gingivalis* was observed in 18% of patients and *Prevotella intermedia* in 42%. *Campylobacter* was observed in 81% and *Neisseria* in 88%. *C. albicans* was observed in only one patient. No significant differences were found between both groups. Part of the child population studied had anaerobic bacteria. There is no clear association between the presence of periodontopathogens and SSC.

## 1. Introduction

Dental caries is one of the most common diseases in children. If the carious lesion is extensive, the affected teeth can be treated with stainless steel crowns (SSCs) [1].

The oral microbiota is the group of microorganisms that make up the oral ecosystem. It is estimated that there are at least 700 species, including bacteria, fungi, viruses, archaea, and protists. Bacterial species play an important role in the maintenance of oral health and in the etiology of oral diseases in humans. Under balanced conditions (eubiosis), proper physiological function and maintenance of health is ensured. However, when the microbial balance is disturbed (dysbiosis), it provides a perfect ecosystem for the development of oral diseases such as dental caries and gingivitis/periodontitis. Placing a crown in the oral cavity is enough to create a habitat for the attachment of microorganisms that cause dysbiosis [2,3].

*Streptococcus mutans* is the bacterium most commonly associated with caries. However, the microbiota associated with caries is complex, involving a number of other acidogenic bacteria such as *Actynomyces*, *Corynebacterium*, non-mutans *Streptococcus*, *Veillonella*, and *Capnocytophaga*. With regard to gingival disease, the most important periodontal pathogens in the virulence of the disease are *Porphyromonas gingivalis* and *Prevotella intermedia/Prevotella nigrescens*, *Treponema denticola*, *Tannerella forsythia*, and *Aggregatibacter actinomycetemcomitans*, although others such as *Fusobacterium nucleatum* spp. and *Campylobacter rectus* are also involved.

In relation to periodontal disease, the most commonly involved microorganisms belong to Socransky’s red and orange complexes [4].

Culture analysis methods are used to assess bacterial etiology. Culture is useful to isolate bacterial groups, to study the relationships between them, and to perform susceptibility determinations, but it is often cumbersome and does not allow to represent the total bacterial composition because, on the one hand, the high number of microorganisms present makes it necessary to perform many dilutions to be able to count the colony-forming units per milliliter (cfu/mL) in universal media and, on the other hand and more importantly, only 50–70% of the oral microbiota can be cultured [5].

Therefore, molecular methods such as 16S rRNA detection of bacterial gene fragments are now used in oral cavity samples both for quantification of the total bacterial load using universal primers and for identification of defined species using specific primers, as selection of sequences from highly conserved regions allows accurate identification. In the current literature, there are not many studies evaluating how and when the peri-pathogenic microorganisms first colonize the oral cavity in children. There is also a lack of literature investigating the influence of metal crowns on the microbiota of the pediatric population [6,7].

The aim of this clinical trial was to compare quantitative and qualitative changes in children’s oral microbiota and assess the presence of periodontal pathogens in SSC-treated primary molars compared to healthy primary molars.

## 2. Materials and Methods

This split-mouth clinical trial was carried out on 33 children at the Department of Paediatric Dentistry of the Dental Hospital of the University of Barcelona (HOUB). The protocol of the retrospective case–control open-mouth study was carried out and approved by the Ethics Committee of the Dental School of the University of Barcelona, protocol 40/2021, on 26 October 2021. The sample size was calculated at a 95% confidence level using a Wilcoxon signed-rank test with a power of 80% to stimulate the data following the uniform Laplace and normal distributions [8]. The significance level for the results was set at 5%. For multiple comparisons or multiple tests, the *p*-values were adjusted to control the false discovery rate (FDR). This study was conducted between February and May 2023. The aim and procedure of the study were explained to parents or legal guardians and informed consent was obtained from them in all children included.

Patients included in this study had to meet the following inclusion criteria: they had to be healthy patients (ASA I and II) between the ages of four and ten, with at least one primary molar restored with a stainless-steel crown (D or E) and another healthy primary molar (D or E). Patients were also screened to ensure that they had not taken any antibiotics in the three weeks prior to sample collection and had not had a professional dental cleaning in the previous month. Exclusion criteria for this study were patients with ASA III, patients whose parents or guardians did not give consent, patients with psychological and motor difficulties due to difficulty in removing plaque, or uncooperative patients from whom the sample could not be collected. In the end, 33 patients were selected (18 boys and 15 girls). Their ages ranged from 4 to 10 years, with a mean age of 6 years. All subjects were clinically examined by the principal investigator prior to sample collection.

Gingival plaque accumulation was scored using a four-category scale as follows: (0) no plaque/debris on inspection and probing, (1) thin layer of plaque visible only after probing, (2) layer of plaque covering the sulcus and gingival areas of the crown but not filling the interdental space, and (3) thick layer of plaque already visible.

We evaluated clinically the gingival status of the metal-crowned primary molars and the healthy primary teeth. The plaque index was evaluated using O’Leary index using dental plaque revealer in liquid format. Löe and Silness gingival index was used, in which the inflammation of each of the four gingival areas (buccal, mesial, distal, and lingual) was assessed and a value was assigned from zero to three: 0 = normal gum, 1 = mild inflammation: color change and slight edema without hemorrhage on probing, 2 = moderate inflammation: redness, edema, and shine with hemorrhage on probing, 3 = intense inflammation: intense redness and edema with a tendency to spontaneous hemorrhage. Probing depth was measured and bleeding after probing was recorded 20 s after probing (present = 1, absent = 0) at 4 points (vestibular, mesial, distal, and lingual) to evaluate gingival health in specific areas of the teeth. All measurements were taken using sterile material [9]. Subgingival plaque samples were collected with sterile curettes and immediately transferred to an Eppendorf tube containing 1 mL of a transport medium specific for anaerobic bacteria, called ‘reduced transport fluid’ (RTF). The samples were then taken to the Microbiology Laboratory of the University of Barcelona, where they were processed in less than 24 h and the microbiological study was carried out.

The sample was vortexed for 1 min and serially diluted to 10^3^ with PBS. A total of 0.1 mL (100 μL) of this diluted sample was inoculated into Petri dishes containing microbiological media, horse blood agar and selective solid media. Several media were used: a selective medium for streptococci, horse blood agar with nalidixic acid and colistin (CNA), media for anaerobes, fastidious anaerobe agar (FAA), and two plates of Dentaid agar. Samples were plated on the solid media using the Digralsky handle. The plates of CNA and Dentaid 1 plates were incubated at 37 °C for 48 h in a 5% CO_2_ atmosphere. The plates of FAA and Dentaid 2 plates were incubated for 7 days at 37 °C under anaerobic conditions (Don Withley DG 250 chamber, Don Whitley Scientific, Bingley BD16 2NH, United Kingdom, 10% CO_2_, 10% H_2_, and 80% N_2_). At the end of the incubation period, colony characteristics were examined and the mean colony-forming units (CFU/mL) were counted to determine the total number of bacteria in both samples from each patient. Counts were performed after 48 h or 7 days of incubation for the microaerophilic and anaerobic bacteria, respectively. DNA extraction from pure bacterial cultures was performed using the QIAamp DNA Mini Kit (QIAGEN, Valencia, CA, USA), according to the manufacturer’s instructions. The concentration and purity of the extracted DNA was determined spectrophotometrically (260/240 nm) using a Nanodrop One (Thermo Scientific, Waltham, MA, USA). DNA samples were then stored at −20 °C until further use.

Real-time PCR was performed using the TaqMan assay system. Specific primers designed to amplify regions of the *16S ribosomal RNA* (*rRNA*) gene (Invitrogen Custom, Thermo Scientific, Waltham, MA, USA) were used, with a final concentration of 25 μM. The specific fluorescent probes were used at a final concentration of 100 pmol/μL (Sigma-Aldrich, St. Louis, MO, USA). The sequence of the primers and probe used was as follows: Total Bacteria F [5′ to 3′] TCCTACGGGAGGCAGCAGCAGCAGT, Total Bacteria R [5′ to 3′]GCACTACCAGGGTATCTAAYCCTGTT, Total Bacteria R [5′ to 3′]GCACTACCAGGGTATCTAAYCCTGTT, and probe: Universal Bacteria [6FAM]CGTATTACCACCGCGGGGCTGCTGGCAC[TAM].

### Statistical Analysis

A significance level of 95% was required to assess the significance of the results. *p*-values were adjusted using the Holm method to control for false discovery rate (FDR) for multiple comparisons or multiple testing. As there were repeated samples from each patient (restored and unrestored teeth), the model included a covariate to indicate sample type, as well as a random effect for patients (linear mixed model). When numerical variables with low prevalence were used, a negative binomial distribution was employed to model the excess zeros (inflated linear mixed model).

All analyses were performed using R v 4.3.2. A descriptive analysis of the quantitative variables (mean and standard deviation) was performed. Two tests were performed: first, a Shapiro–Wilks test to see if the values were not significantly different (95% confidence level) from those generated by a normal distribution. If this test was not rejected, a paired Student’s *t*-test for difference in means was calculated; otherwise, a Wilcoxon signed rank test was performed. In practice, for the three cases, the reported *p*-value came from the latter test. All this was performed using R packages. The descriptive tables were generated using the compare groups and create table functions of the R package compare groups. The null hypothesis for this paired nonparametric test (Wilcoxon) is that the distribution of differences between samples (healthy vs. treated) of individuals is symmetric and centered on zero.

## 3. Results

This study involved a total of 33 children whose guardians agreed to participate and from which a total of 66 samples were collected (two from each patient).

**Figure 1:**
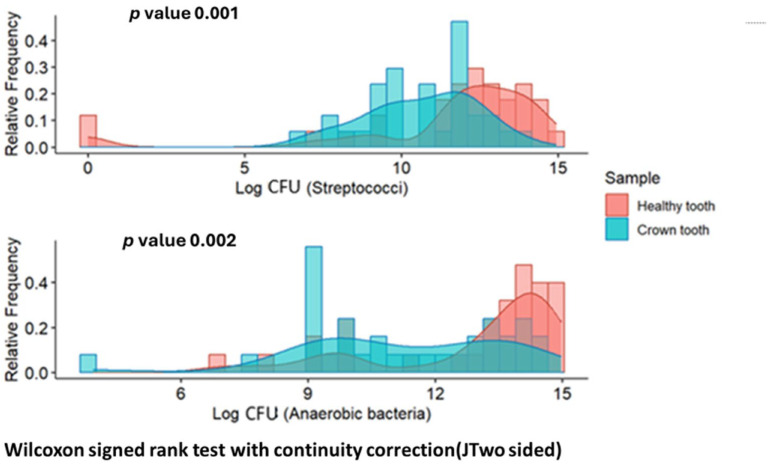
Histogram of frequencies of colony-forming units/mL (CFU/mL) of streptococci and anaerobic bacteria according to type of sample (healthy tooth vs. crown tooth). The comparison of CFU/mL of bacteria between sample types is of their prevalence.

Significant differences (*p* < 0.01) were observed in the distribution of CFU/mL of streptococci and anaerobic bacteria between both groups (always higher in the healthy tooth sample). Figure 1 shows the frequency distributions of the number of CFU of both types in each group.

In terms of the total number of samples analyzed, *P. gingivalis* was observed in 18% of patients and *P. intermedia* in 42% of patients. Campylobacter was observed in 81% and Neisseria in 88% of patients. *C. albicans* was observed in only one patient. *A. actinomycetemcomitans* was not observed in any patient. No differences were observed in the total CFU counts of *P. gingivalis*, *P. intermedia*, *Campylobacter*, and spp. between healthy and restored tooth samples.

No significant differences were observed in the total 16S PCR bacterial load according to sample type. There was a positive correlation between 16S PCR bacterial load and bacterial count per culture in both studied groups (Spearman 0.5688, CI: 0.3148–0.7327, *p* (7.219 × 10^−6^)).

**Figure 2:**
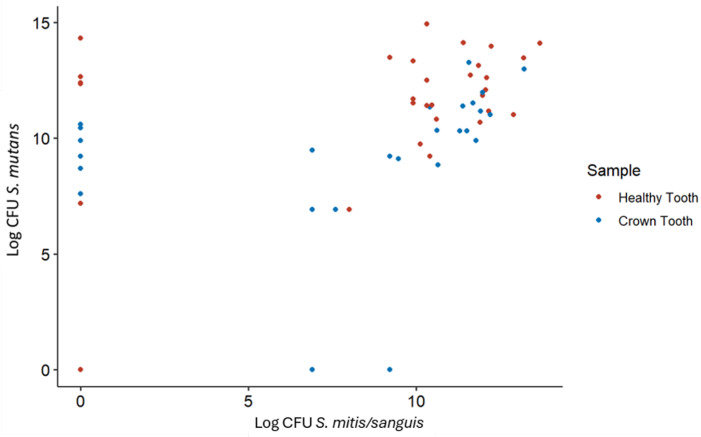
Bivariate distribution of bacterial counts (Log CFU/mL *S. mitis*/*sanguis* vs. Log CFU/mL *S. mutans*). (cor 0.360 *p* 0.00004 Kendall method).

Regarding the presence or absence of the different bacterial species according to the type of sample, no significant differences were observed in any of the bacteria investigated. On the other hand, there are significant differences (*p* < 0.005) in the prevalence of certain combinations (*S. mutans* and *S. salivarius*, *S. mitis*/*sanguis* and *P. gingivalis*, and *S. mutans* and *P. gingivalis*). Data are shown in Figure 2 and Table 1, Table 2 and Table 3. 

**Figure 3:**
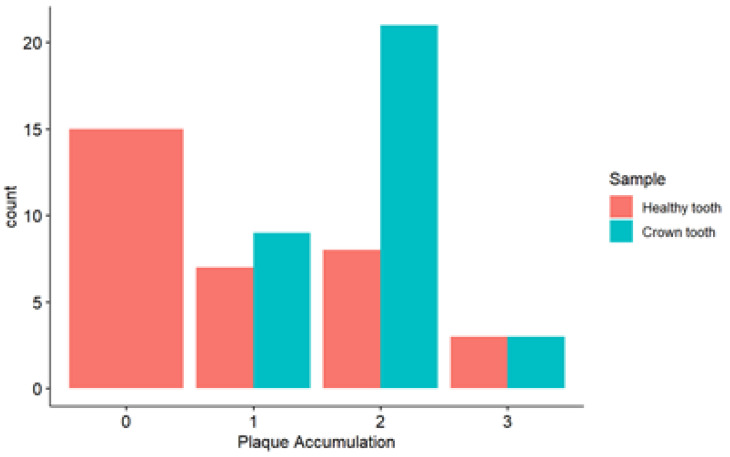
Distribution of the variable plaque accumulation (measured using O’Leary index) by sample type. Sample not treated = Healthy tooth; sample with crown = Treated tooth.

In the analysis of plaque accumulation in healthy teeth, the majority of cases (15/33) are concentrated in the lower level (0). For the tooth with SSC, the majority (21/33) were concentrated in level 2 (Figure 3).

**Figure 4:**
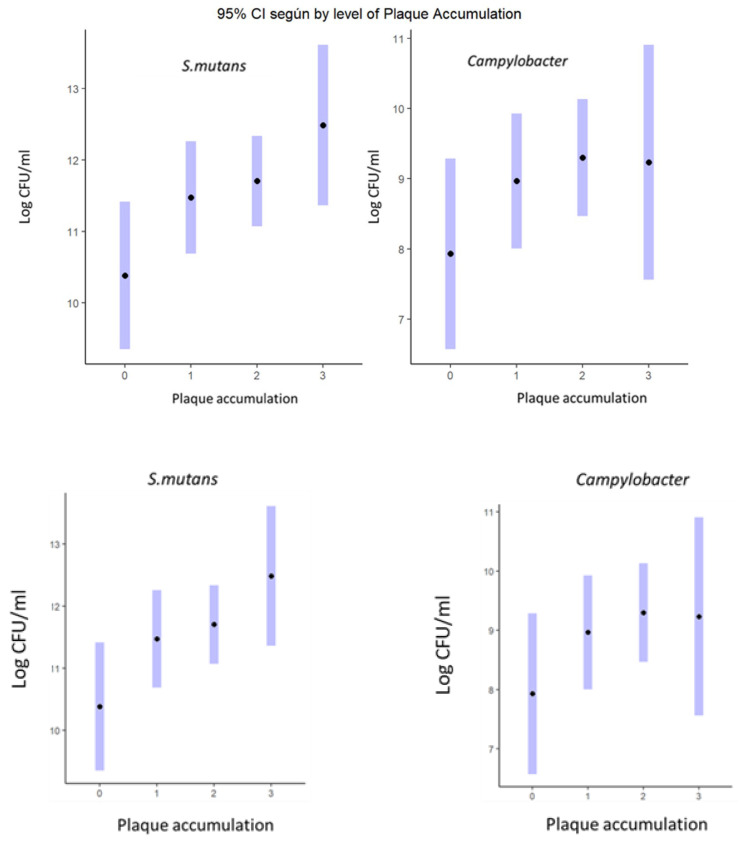
Plaque accumulation (O’Leary index) significant differences in coefficients per bacterium (Log of colony-forming units/mL) (95% significance level). The black dots are the adjusted means (zero-inflated model).

Regarding the relationship between plaque accumulation and the type of bacteria present, in the case of *P. gingivalis*, it could not be evaluated due to the low number of isolates and, in the case of *P. intermedia*, no significant differences were found but differences were found in *S. mutans* (*p* < 0.001) and *Campylobacter* spp. (*p* < 0.05); see Figure 4.

## 4. Discussion

When dysbiosis or disturbance of the microbial balance occurs, diseases such as dental caries or periodontal diseases such as gingivitis and periodontitis appear.

Dental caries disease is very prevalent in children. On the other hand, the most common periodontal disease in children is gingivitis and its inflammatory reaction will depend on the microbial biofilm and bacterial aggression of the host. Periodontitis is usually present in adulthood. The microbiota is established in childhood and changes throughout life [10].

The presence of streptococci favors the formation of dental plaque, acting as early colonizers and consequently favoring gingival inflammation. In the present study, a greater presence of streptococci was observed in teeth without crowns. However, more plaque accumulation was observed in teeth with crowns. We assume that other bacteria would act on the surface of the crowns, facilitating the formation of the biofilm. Streptococci play an important role in biofilm formation, but it is a complex process involving many other bacteria, which explains the results. These differences could be due to the fact that the material of the metal crowns hinders the adhesion of streptococci and favors the formation of a biofilm with a different composition. These results agree with the study of Bin AlShaibah et al. in which they observed that *S. mutans* adhesion was lower in SSC compared to other crowns and associated the increase in *S. mutans* with a higher oral hygiene index and gingival index [10]. The study by Hamza B. et al. evaluated the initial adherence and biofilm formation on different materials; they observed that SSC had a lower initial bacterial adherence; however, there were no significant differences between the materials studied in terms of biofilm formation [11].

In our study, we observed a linear correlation between *S. mutans* and *S. sanguinis/S. mitis*, probably because there is no antagonism between them. Pathogenic and nonpathogenic streptococci coexist in the oral cavity without causing more problems than dental caries, but it should not be forgotten that both can cause endocarditis at the systemic level.

As other authors have pointed out, it is likely that gingivitis occurring in proximity to restorative materials is caused by bacterial plaque rather than by direct mechanical irritation from the materials themselves [3,12].

Referring to the periodontium, several studies have observed the presence of periodontopathogen bacteria in the pediatric population in different proportions and ages [6,13,14].

Belduz Kara and Yılmaz [15] comparing the oral hygiene and gingival health of restored teeth with SSCs found that control teeth had better periodontal health than restored teeth. However, their study found that clinically detectable plaque on the restored teeth was comparable to that on the control teeth. They also found that periodontal health deteriorated with time regardless of the type of crown used. Their results were attributed to oral hygiene compliance.

Prabhu et al. found worsening gingival status of molar teeth restored with SSCs over time compared to control teeth. However, they observed less plaque and debris accumulation in SSCs due to decreased plaque adherence to the smooth surface [16].

In the present study, no statistically significant differences in periodontopathogen bacteria *P. gingivalis* and *P. intermedia* were observed between the two groups studied and *A. actinomycetemcomitans* appeared in none of them. Periodontopathogens were present in 42% of the samples from the pediatric population studied (*Campylobacter* in 81%). This agrees with the study by Takahashi K. et al., who found periodontopathogens in the pediatric population. They observed that there were no significant differences in children without teeth and the ones with primary teeth. However, they did observe differences in the mixed and permanent dentition [7]. In their study, Ali S. Alghamdi et al. found no statistically significant differences in the microbiota between children with gingivitis and those without periodontal pathology [17].

Therefore, the presence of metal crowns alone may not be a determining factor in the occurrence of periodontopathogen bacteria, but a combination of other factors plays a major role in it.

In the study by Takahashi K. et al., they concluded that the periodontal status of parents significantly influences the composition of their children’s microbiome, with a positive correlation between the presence of periodontopathogens between parents and children [7]. This agrees with the study by Reis A. et al., in which they concluded that the mixed dentition phase is most associated with a dysbiotic microbiome with the presence of pathogens in cases where the parents have periodontal disease [13].

In the studies of Motoc G. et al., they concluded that the identification of periodontopathogens was associated with age, body mass index, diet in the first 6 months of life, and salivary pH. They revealed a notable influence of dietary habits and diet on the presence and intensity of specific periodontal pathogens such as high sugar consumption leading to an increase in bacteria such as *Capnocytophaga* spp. [18].

Comparative studies have shown that stainless-steel crowns produce more successful clinical results than conventional composite restorations. This may be because stainless-steel crowns provide an excellent seal, which is an important factor in achieving successful restorative treatment results [19].

In our study, no significant quantitative differences in total CFU measured by 16S-PCR were observed between the two samples.

Our study has several limitations, including the short time frame of three months, which may not reveal long-term changes in the microbiota. It should also be noted that no sequencing studies have been carried out and that we have only analyzed the part of the oral microbiota that grows in culture and for which specific primers for molecular detection are available.

## 5. Conclusions

In the population studied, no significant qualitative or quantitative differences were found of total cfu/mL counts between teeth with SSC and healthy teeth, with the exception of *S. mutans*, which was found in lower quantities in the samples with crowns. A part of the child population studied had anaerobic bacteria. However, there is no clear association between the presence of periodontopathogens and SSC. Other factors influencing the occurrence of these bacteria should be evaluated.

It would be interesting to study the chronology and evolution of the formation of the biofilm on the stainless-steel surfaces of the crowns. It would also be interesting to study the quantitative composition of the biofilm, which appears thicker on these surfaces.

Ideally, a future study should be carried out that compares the crowns of different materials and suppliers in terms of roughness, composition, and adhesiveness.

Further studies are needed to evaluate the oral microbiota in children as well as its changes in the presence of restorative treatments such as SSCs.

Further research is needed to understand the role of microbiological changes in children’s oral health that will influence adult oral health.

## Figures and Tables

**Table 1:** Comparative colony-forming units per mL (CFU/mL) counting in culture and data obtained by real-time PCR (corresponding to bacterial load). Sample not treated = Healthy tooth; sample with crown = Treated tooth. Statistics for non-normal distribution: median and IQR: interquartile ranges.

Pacient	Bacterial Load Concentration Pcr (UFC/mL) Healthy Teeth	Bacterial Load Concentration PCR (UFC/mL) Treated Teeth	Bacterial Load Concentration Counting (UFC/mL) Healthy Teeth	Bacterial Load Concentration Counting (UFC/mL) Treated Teeth
1	4.80 × 10^7^	9.84 × 10^7^	2.80 × 10^3^	4.00 × 10^4^
2	2.31 × 10^8^	1.45 × 10^8^	2.50 × 10^6^	8.30 × 10^5^
3	1.08 × 10^8^	1.72 × 10^8^	8.20 × 10^5^	6.40 × 10^5^
4	3.49 × 10^7^	1.75 × 10^6^	2.04 × 10^7^	1.22 × 10^4^
5	5.16 × 10^7^	2.29 × 10^7^	2.20 × 10^6^	1.30 × 10^5^
6	1.13 × 10^5^	2.28 × 10^5^	1.00 × 10^3^	2.00 × 10^3^
7	7.95 × 10^6^	1.69 × 10^8^	1.10 × 10^5^	5.20 × 10^5^
8	1.40 × 10^9^	2.69 × 10^4^	1.06 × 10^6^	7.80 × 10^5^
9	0	0	1.04 × 10^6^	1.48 × 10^6^
10	0	0	2.08 × 10^6^	6.00 × 10^4^
11	7.25 × 10^4^	0	9.00 × 10^3^	1.62 × 10^6^
12	0	1.33 × 10^6^	2.16 × 10^6^	2.10 × 10^5^
13	3.47 × 10^8^	6.96 × 10^7^	1.25 × 10^6^	5.00 × 10^5^
14	2.65 × 10^10^	2.86 × 10^10^	2.62 × 10^6^	1.22 × 10^6^
15	6.18 × 10^9^	1.10 × 10^10^	5.40 × 10^5^	1.00 × 10^4^
16	7.14 × 10^7^	1.80 × 10^8^	2.00 × 10^6^	5.00 × 10^4^
17	3.50 × 10^8^	2.75 × 10^6^	1.63 × 10^6^	2.00 × 10^4^
18	0	1.30 × 10^7^	4.30 × 10^5^	3.00 × 10^5^
19	2.88 × 10^9^	2.88 × 10^7^	1.58 × 10^6^	3.00 × 10^4^
20	6.24 × 10^8^	3.81 × 10^9^	2.50 × 10^6^	6.20 × 10^5^
21	3.56 × 10^9^	1.42 × 10^7^	1.36 × 10^6^	1.00 × 10^4^
22	8.25 × 10^9^	8.36 × 10^8^	1.40 × 10^6^	1.00 × 10^5^
23	1.62 × 10^8^	2.40 × 10^7^	1.18 × 10^6^	1.32 × 10^6^
24	9.21 × 10^5^	4.11 × 10^5^	2.00 × 10^4^	1.00 × 10^4^
25	1.84 × 10^8^	1.67 × 10^4^	1.42 × 10^6^	2.00 × 10^4^
26	2.82 × 10^5^	2.86 × 10^7^	5.50 × 10^5^	5.00 × 10^5^
27	5.25 × 10^7^	3.79 × 10^7^	3.00 × 10^5^	2.20 × 10^6^
>28	1.84 × 10^5^	7.98 × 10^5^	1.00 × 10^4^	1.00 × 10^4^
29	8.94 × 10^5^	9.91 × 10^5^	5.20 × 10^5^	1.00 × 10^4^
30	2.85 × 10^8^	6.79 × 10^6^	2.48 × 10^6^	2.00 × 10^4^
31	3.80 × 10^7^	2.68 × 10^6^	3.20 × 10^6^	1.00 × 10^4^
32	7.37 × 10^7^	1.99 × 10^6^	8.40 × 10^5^	1.00 × 10^4^
Median	6.20 × 10^7^	1.36 × 10^7^	1.12 × 10^6^	8.00 × 10^4^
IQR	3.47 × 10^8^	1.09 × 10^8^	1.70 × 10^6^	6.13 × 10^5^

**Table 2:** Distribution of the prevalence of different bacteria according to sample type. Sample not treated = Healthy tooth; sample with crown = Treated tooth. Data obtained from bacteriological cultures.

Bacteria	Healthy Tooth	Treated Tooth
*S. mutans*	31 (94%)	31 (94%)
*S. salivarius*	6 (18%)	7 (21%)
*S. mitis/sanguinis*	24 (73%)	22 (67%)
*P. gingivalis*	2 (6%)	4 (12%)
*P. intermedia*	12 (36%)	9 (27%)
*Campylobacter*	23 (70%)	22 (67%)
*Neisseria* spp.	22 (67%)	19 (58%)

**Table 3:** Combinations of bacteria for which their difference in prevalence depending on the type of sample is significant (95% significance level). Data obtained from bacteriological cultures.

Combination	*p* Value
*S. mutans* + *P. gingivalis*	0.00002
*S. mutans* + *P. salivarius*	0.00159
*S. mitis/sanguinis* + *P. gingivalis*	0.00199

## Data Availability

The original contributions presented in the study are included in the article, further inquiries can be directed to the corresponding author.
